# Prenatal Di‐methoxyethyl phthalate exposure impairs cortical neurogenesis and synaptic activity in the mice

**DOI:** 10.1111/bpa.13221

**Published:** 2023-10-30

**Authors:** Moon Yi Ko, Heejin Park, Sun‐Hwa Chon, Byoung‐Seok Lee, Sin‐Woo Cha, Sung‐Ae Hyun, Minhan Ka

**Affiliations:** ^1^ Department of Advanced Toxicology Research Korea Institute of Toxicology Daejeon Republic of Korea; ^2^ Collage of Veterinary of Medicine Jeonbuk National University Jeonju Republic of Korea

**Keywords:** di‐methoxyethyl phthalate (DMEP), hyperactive behaviors, neurogenesis, synapse

## Abstract

Di‐methoxyethyl phthalate (DMEP) is a well‐known environmentally prevalent endocrine disruptor and may be associated with neurodevelopmental disorders including attention deficit/hyperactivity disorder and intellectual disability. However, the regulatory mechanisms leading to these neurodevelopmental disorders are still poorly understood. Here, we demonstrate that prenatal DMEP exposure causes abnormal brain morphology and function in the mice. DMEP (50 mg/kg) was chronically administered to pregnant mice orally once a day starting on embryonic day 0 (E0) to breast‐feeding cessation for the fetus. We found that prenatal DMEP exposure significantly reduced the number of neurons in the parietal cortex by impairing neurogenesis and gliogenesis during the developing cortex. Moreover, we found that prenatal DMEP exposure impaired dendritic spine architectures and synaptic activity in the parietal cortex. Finally, prenatal DMEP exposure in mice induces hyperactivity and reduces anxiety behaviors. Altogether, our study demonstrates that prenatal DMEP exposure leads to abnormal behaviors via impairment of neurogenesis and synaptic activity.

## Graphical abstract

- Prenatal DMEP exposure can lead to abnormal neurogenesis and astrogenesis in brain development.
- Prenatal DMEP exposure changes the gene expression profile during cortical development.
- Prenatal DMEP exposure leads to synaptic dysfunction in the cerebral cortex by affecting dendritic spine and synaptic formations.
- Prenatal DMEP exposure results in abnormal behaviors.

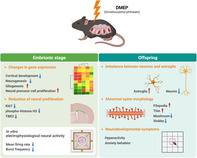

## 1 INTRODUCTION

Phthalates are environmental pollutants mainly used as plasticizers for increased flexibility, transparency, durability, and longevity of plastic products [1, 2]. Phthalates are commonly found in many consumer products including pharmaceuticals, food packaging, paints, furniture, cosmetics, and toys [2], and they can enter the human body mainly via oral, inhalation or dermal contact [3, 4, 5]. After the human body is exposed to phthalates, they are not easily degraded and can be detected in human serum and plasma [6, 7]. Moreover, phthalates can easily through the placental barrier reaching fetal circulation and affect developing fetuses [8, 9].

Recently, many studies showed strong evidence of the relationship between prenatal phthalate exposure and the risk of attention‐deficit/hyperactivity disorder (ADHD) [10, 11, 12]. Prenatal exposure to di‐2‐ethylhexyl phthalate (DEHP) induces anxiety and memory loss via activation of neuronal degeneration in the mice [13]. Perinatal exposure to DEHP affects depression‐like behaviors via the downregulation of androgen and estrogen receptors in the mice [14]. Moreover, pubertal exposure to DEHP affects social behaviors via the regulation of dopamine receptor D2 in adult mice [15]. Additionally, exposure to dibutyl phthalate (DBP) induces neuronal apoptosis, synaptic dysfunction, and neurobehavioral problems in rodents [16, 17, 18]. Furthermore, DBP exposure leads to impaired adult neurogenesis and learning and memory dysfunctions through neuroinflammation in adult mice [19]. Epidemiological evidences have shown that prenatal exposure to phthalates is related to decreased IQ scores and adverse mental and motor development in children [20, 21, 22]. Thus, based on these findings, the neurological system can be considered an important target of phthalates. Moreover, there are many reports of effects on neurotoxicity caused by phthalates, such as DEHP or DBP; however, the correlation between behavioral and neurological dysfunction caused by di‐methoxyethyl phthalate (DMEP) and the mechanisms underlying these dysfunctions are poorly understood. We hypothesized that prenatal exposure to DMEP would affect brain development in mice. The lowest observable adverse effect levels (LOEALs) of critical effects caused by DMEP were 100 mg/kg for hematological changes and 50 mg/kg for reproductive toxicity with a decrease in testosterone [23]. In addition, DMEP showed developmental effects such as reduced pup weight at 60 mg/kg doses [24]. Therefore, to examine the effect of DMEP during brain, pregnant mice were exposed to DMEP at a dose of 50 mg/kg, the lowest LOEAL value showing major toxicity.

In this study, we consequently observed abnormal synaptic formation and transmission in DMEP‐exposed neurons. Finally, this abnormal cortical morphogenesis and synaptic activity led to hyperactivity and reduced anxiety in prenatal DMEP‐exposed mice. Our results may provide a molecular mechanism to better understand DMEP‐induced neurodevelopmental defects.

## 2 MATERIALS AND METHODS

### 2.1 Chemical

DMEP (>96.0% [GC]) was purchased from Tokyo Chemical Industry (catalog number: P0965) and dissolved in saline.

### 2.2 Animals and sample collection

Female mice (C57BL/6N) were mated with male mice under the condition of the temperature (23 ± 3°C) and humidity (30%–70%) with standard rodent chow and water available ad libitum and were maintained on a 12 h light/dark cycle (lights on at 8:00 a.m.). Detection of vaginal plug was designated as embryonic day 0 (E0). Pregnant mice were administered by either DMEP (50 mg/kg) or saline (control) orally once a day from E0 to the cessation of breast‐feeding the fetus. Both experimental groups used seven pregnant mice. To perform embryonic experiments, three pregnant mice were sacrificed at E14.5 stage. Seven embryos were used for cortical development experiments. Litters were culled to a maximum of seven pups and breastfed until postnatal day 21. To minimize possible littermate effects, two or three male pups from each dam were randomly selected for early postnatal day (P3) experiments. The body weight and brain weight were measured at P3 stage. Other pups of both groups were weaned at P21 and separated by sex. At postnatal 56 stage, seven male pups were used for behavior studies and then sacrificed for the analysis of dendritic spine and synaptic formation experiments. Mice handling and experiments performed according to our animal protocol approved by the Institutional Animal Care and Use Committee at the Korea Institute of Toxicology (approval numbers 20‐10231).

### 2.3 Immunoblotting

Immunoblotting was performed according to the protocol described in our previous publications [25, 26]. Cortical lysates from the E14.5 and P3 stage was extracted using radioimmunoprecipitation assay buffer (89900, Thermo Fisher Scientific). Twenty microgram of total proteins was subjected to sodium dodecylsulphate–polyacrylamide gel electrophoresis in 8% or 10% polyacrylamide gels at 100 V for 2 h. The gel was electro‐transferred onto a polyvinylidene difluoride membrane (88518, Thermo Fisher Scientific). The membrane was incubated with the following primary antibodies at 4°C overnight. Primary antibodies used for immunoblotting are as follows (Table 1): rabbit anti‐Hes5, rabbit anti‐SOX2, mouse anti‐NeuN, rat anti‐GFAP, and mouse anti‐β‐actin. Then, corresponding secondary antibodies conjugated to HRP (65‐6120, 62‐6520, and 31470, Thermo Fisher Scientific) and the membranes were reacted with enhanced chemiluminescence reagents (34075, Thermo Fisher Scientific). The protein bands were visualized by an ImageQuant LAS 500 system (GE Healthcare). The expression levels of proteins were normalized to β‐actin levels.

**TABLE 1:** List and dilutions of primary antibody.

Antibodies	Conc.	Company	Catalog no.
Mouse anti‐NeuN	1:1000	Abcam	ab104224
Rat anti‐GFAP	1:1000	Thermo Fisher Scientific	#13‐0300
Rat anti‐KI67	1:1000	Thermo Fisher Scientific	11‐5698‐82
Mouse anti‐phospho‐Histone H3	1:1000	Thermo Fisher Scientific	MA5‐15220
Rabbit anti‐Tbr2	1:1000	Abcam	ab23345
Rabbit anti‐Hes5	1:5000	Abcam	ab25374
Rabbit anti‐SOX2	1:5000	Abcam	ab97959
Chicken anti‐GFP	1:1000	Thermo Fisher Scientific	A10262
Mouse anti‐VGLUT	1:1000	Synaptic Systems	135001
Mouse anti‐VGAT	1:1000	Synaptic Systems	131011
Mouse anti‐β‐actin	1:5000	Thermo Fisher Scientific	A5316

### 2.4 Immunostaining

Immunostaining was performed according to the protocol described in previous publications [27, 28]. Briefly, brain tissue was perfused using 4% paraformaldehyde and dehydrated using 30% sucrose solution for 48 h. Brain tissue was embedded in low melting point agarose (17856, Thermo Fisher Scientific). Then brain tissue sectioned at 80 μm thickness using VT1200 vibratome (Leica). Cultured neurons were fixed using 4% paraformaldehyde for 10 min and permeabilized using 0.5% Triton X‐100 for 10 min at room temperature (RT). Then, the perfused brain tissue or fixed cultured neurons were blocked with 10% normal goat serum for 90 min at RT and incubated at 4°C overnight with primary antibodies. Primary antibodies used for immunostaining (Table 1): mouse anti‐NeuN, rat anti‐GFAP, rat anti‐KI67, mouse anti‐phospho‐Histone H3, rabbit anti‐TBR2, chicken anti‐GFP, mouse anti‐VGLUT, and mouse anti‐VGAT. The next day, the corresponding secondary antibodies (Thermo Fisher Scientific) were reacted with the incubated cells at RT for 2 h. Nuclei were stained with 4,6‐diamino‐2‐phenylindole (DAPI, Sigma). Images were captured using an Olympus FV3000 microscope and Olmpus software (Olympus Life Science). We counted NeuN‐, GFAP‐, KI67‐, phospho‐H3‐, or TBR2‐positive cells in a field of 0.2 or 0.4 mm^2^ throughout the rostrocaudal extent of the VZ/SVZ, IZ or the cortical plate of the parietal cortex.

### 2.5 Analysis of dendritic spine architecture

Cortical neurons from E14.5 mice were cultured for 10 days and then transfected with a plasmid encoding the green fluorescent protein (GFP). Neuronal transfection was carried out following the procedure described in previous publication [29]. DNA constructs were transfected into the attached cells using lipofectamine according to the protocol provided by the manufacturer. Following 4 days of culturing, cortical neurons were exposed to DMEP or 0.1% DMSO for 24 h. Dendritic spines were then assessed by immunostaining with GFP. For classification of dendritic spine types, the following criteria were used: Filopodia are typically longer (>2 μm) without a clear head; thin spines have a thin and long neck (>1 μm) and a small head; mushroom spines have a short and narrow neck (<1 μm) and a large head (>0.6 μm), whereas the stubby spines have a head but no neck. For identification of synapses in cortical neurons, cultured neuron synapses were immunostained with antibodies that are specific to synaptic markers, namely vesicular glutamate transporter (VGLUT; also known as SLC17A; excitatory synapses) and vesicular GABA transporter (VGAT; also known as SLC32A1; inhibitory synapses). For the analysis of spine density, imaging of secondary dendrites of cultured cortical neurons was performed (z‐stack thickness of 0.5 μm) using an Olympus FV3000 confocal laser scanning microscope (Olympus Life Science) equipped with a UPLSAPO 40 × 2/0.95 N.A. 1.4) objective lens.

### 2.6 Golgi staining

Golgi staining was performed using the FD Rapid GolgiStain Kit (PK401A, FD NeuroTechnologies, Inc.) according to the reported protocol [30, 31]. Briefly, whole brains were placed in impregnation solution (a mixture of solution A and solution B) and stored at RT in the dark for 7 days. Then, whole brains were placed in solution C and stored at RT in the dark for 5 days. Next, whole brains were cut coronally at a 200 μm thickness using a VT1200 vibratome (Leica). Brain slices were incubated with staining solution. After rinsing, brain slices were gradually dehydrated with 50%, 75%, 95%, and 100% ethanol. Next, brain slices were cleared with xylenes and mounted with Permount solution (SP15‐100, Thermo Fisher Scientific). For the analysis of dendritic spine density, imaging of secondary dendrites of apical dendrites of pyramidal neurons of II–III cortical layer was performed (z‐stack thickness of 0.5 μm) using an Olympus FV3000 confocal laser scanning microscope (Olympus Life Science) equipped with a UPLSAPO 40 × 2/0.95 N.A. 1.4) objective lens. The number of dendritic spines was determined per micrometer of dendritic length using the ImageJ software (National Instruments of Health).

### 2.7 mRNA sequencing and data analysis

Total RNA was extracted from cortical tissue with TRIzol reagent (15596026, Thermo Fisher Scientific), and complementary DNA (cDNA) was generated from 500 ng of total RNA using oligo‐dT primers containing an Illumina‐compatible sequence. The second strand was synthesized by random primers containing an Illumina‐compatible linker sequence. The double‐stranded cDNA was cleaned by using DNA XP magnetic beads to remove all reaction components. The cDNA was amplified via 15 cycles of polymerase chain reaction (PCR) to add the complete adapter sequences.

The cDNA library was purified from PCR components using DNA XP magnetic beads and quantified using an Agilent 2100 Bioanalyzer (Agilent). Finally, the cDNA library was sequenced into 75 bp single‐end reads using an Illumina NextSeq 500 (Illumina). For data analysis, QuantSeq 3’ mRNA‐Seq reads were quantified using the Bowtie2 tool. Differentially expressed genes (DEGs) were aligned using the coverage tool in the BEDTools toolbox. All differential expression was conducted using the DESeq2 tool in Bioconductor. Heatmaps and volcano plots were generated using Excel‐based DEG analysis tools. KEGG pathway and Gene Ontology (GO) analyses were conducted using DAVID software (http://david.abcc.ncifcrf.gov/).

### 2.8 RT‐qPCR

According to the manufacturer's protocol, total RNA was extracted from cortical tissue using TRIzol reagent (15596026, Thermo Fisher Scientific), and cDNA was generated from 1 μg of total RNA using Maxime™ RT PreMix (25081, Lilif diagnostics). cDNA was amplified by RT‐qPCR using the SsoAdvanced™ Universal SYBR Green Supermix (1725271, Bio‐Rad Laboratories). To perform the quantitative PCR run, CFX Connect Real‐Time PCR Detection System (Bio‐Rad Laboratories) was used with the following cycle parameters: 95°C for 3 min, 40 cycles of 95°C for 10 s, and 54°C for 30 s. The primer sequences for the amplification of Npy, Scn1b, Mdga2, Meis1, Adra2c, Rgs6, Ntm, Hes5, Prox1, and Fzd9 are listed in Table 2. Relative expression levels were calculated by the 2^△△Ct^ method.

**TABLE 2:** List of primer sequences.

Gene symbol	Accession number	Primers	
Npy	NM_023456.3	F	TATCTCTGCTCGTGTGTTTGG
		R	GCCATATCTCTGTCTGGTGATG
Scn1b	NM_011322.4	F	CGACTACGAATGTCACGTCTAC
		R	TGACACGATGGATGCCATATC
Mdga2	NM_207010.2	F	CTCTCTGAAGGGAGGAGGAATA
		R	TCTGGTGACCAAAGGTGATTT
Meis1	NM_001193271.1	F	GTGACGATGATGACCCTGATAA
		R	CAGAAGGGTAAGGGTGTGTTAG
Adra2c	NM_007418.3	F	CTCATGGCCTACTGGTACTT
		R	AGTAGCGGTCCAGACTAATG
Rgs6	NM_015812.4	F	AACACGGACTATGCCATCTATC
		R	CCTCTGGAGTCTTGCTAAGTTT
Ntm	NM_172290.4	F	ACCGCAGTACCATCCTCTAT
		R	CTCATCGTACACATCCACATTCT
Hes5	NM_010419	F	AGCTACCTGAAACACAGCAAA
		R	TGCAGGGTCAGGAACTGTA
Prox1	NM_008937.3	F	CTGCCCTTGATGGCTTAT
		R	CTTGGTCCTCAGACTTGTTGT
Fzd9	NM_010246.1	F	TCCGCGTTGTGTTTCTTCT
		R	GGCCAAGGAGTAGACATTGTAG
Gapdh	NM_001289726.2	F	TGCACCACCAACTGCTTAGC
		R	ATGCCAGTGAGCTTCCCGTT

### 2.9 Multielectrode array system

Primary neuronal culturing was performed as described previously [32]. In brief, cerebral cortices from E14.5 mice were isolated and dissociated with trituration after trypsin/ethylenediamine tetraacetic acid treatment. Then, the cells were plated in poly‐D‐lysine/laminin‐coated multielectrode array plates (M768‐tMEA‐48W, Axion Biosystems) in neurobasal medium supplemented with 1x B27 (A3582801, Gibco), 1% penicillin/streptomycin (15140122, Gibco) and 1× L‐glutamine (25030149, Gibco). After 14 days, the activity of cultured neurons was recorded using the Maestro MEA system (Axion Biosystems, Atlanta, GA, USA). The data were analyzed using Integrated Studio (AxIS) software version 2.5 (Axion Biosystems). Following the baseline recording, cortical neurons were exposed to DMEP, and changes in neuronal activity were evaluated by comparing the baseline activity and postexposure activity.

### 2.10 Behavioral tests

P56 male pups were measured behavioral test such as locomotor activity, elevated plus maze (EPM), and three‐chamber test. All behavioral tests were performed in designated behavioral test room during light cycle. The behaviors were recorded by digital camera (SLA‐3580DN, Samsung Techwin) and analyzed using EthoVision XT 14.0 software (Noldus).

#### 2.10.1 Locomotor activity test

The locomotor activity test was performed according to the protocol described in our previous publication [33]. Briefly, the locomotor activity device was a 71 cm × 86 cm × 56 cm box arena. Each mouse was placed on central area and their moving activity in the arena was recorded for 20 min. Total distance was measured to determine basic locomotor activity. The duration in center and periphery area were measured to determine anxiety behavior.

#### 2.10.2 Elevated plus maze test

The EPM test was performed according to the protocol described in our previous publication [34]. Briefly, the EPM includes two open arms (25 cm × 5 cm) on opposite sides and two closed arms (25 cm × 5 cm × 15 cm) on opposite sides connected by a central square (5 × 5 cm) to form a plus‐sign‐shaped apparatus. The EPM was located at a height of 50 cm above the floor. Each mouse was placed on central area facing the open arms and their moving activity in the arms was recorded for 10 min.

A mouse was placed on the central platform, facing the same open arm, and allowed to roam freely for 10 min. The total time spent on open and closed arms was recorded.

#### 2.10.3 Three‐chamber test

The three‐chamber test was performed according to the protocol described in our previous publication [33]. Briefly, the three‐chamber device is a rectangular apparatus (60 cm × 40 cm × 25 cm) with three equal‐sized compartments (20 cm × 40 cm × 25 cm) (catalog #46503, Ugo Basile). After habituation, the test mouse was placed in the center compartment with the entrances to the two connecting chambers blocked. An unfamiliar mouse designated “novel stranger” was placed in a wire cup in the side compartment. An empty wire cup was placed inside the other side compartment. Then, the openings to the flanking two compartments were opened, and the test mouse was allowed to explore the three chambers for 10 min. Time spent by the test mouse sniffing each compartment was recorded during 10‐min period to test sociability behavior.

### 2.11 Statistical analysis

All data are expressed as the means (±) standard error of the mean (SEM). Data were determined by two‐tailed unpaired Student's *t*‐test for two‐population comparison and one‐way or two‐way analysis of variance (ANOVA) followed by the Bonferroni post hoc test for multiple comparisons. Statistical analysis was performed using Graph Pad Prism 8 software (GraphPad). Statistically significance was set at *p* < 0.05.

## 3 RESULTS

### 3.1 Prenatal DMEP exposure affects proliferating cells during cortical development

**FIGURE 1:**
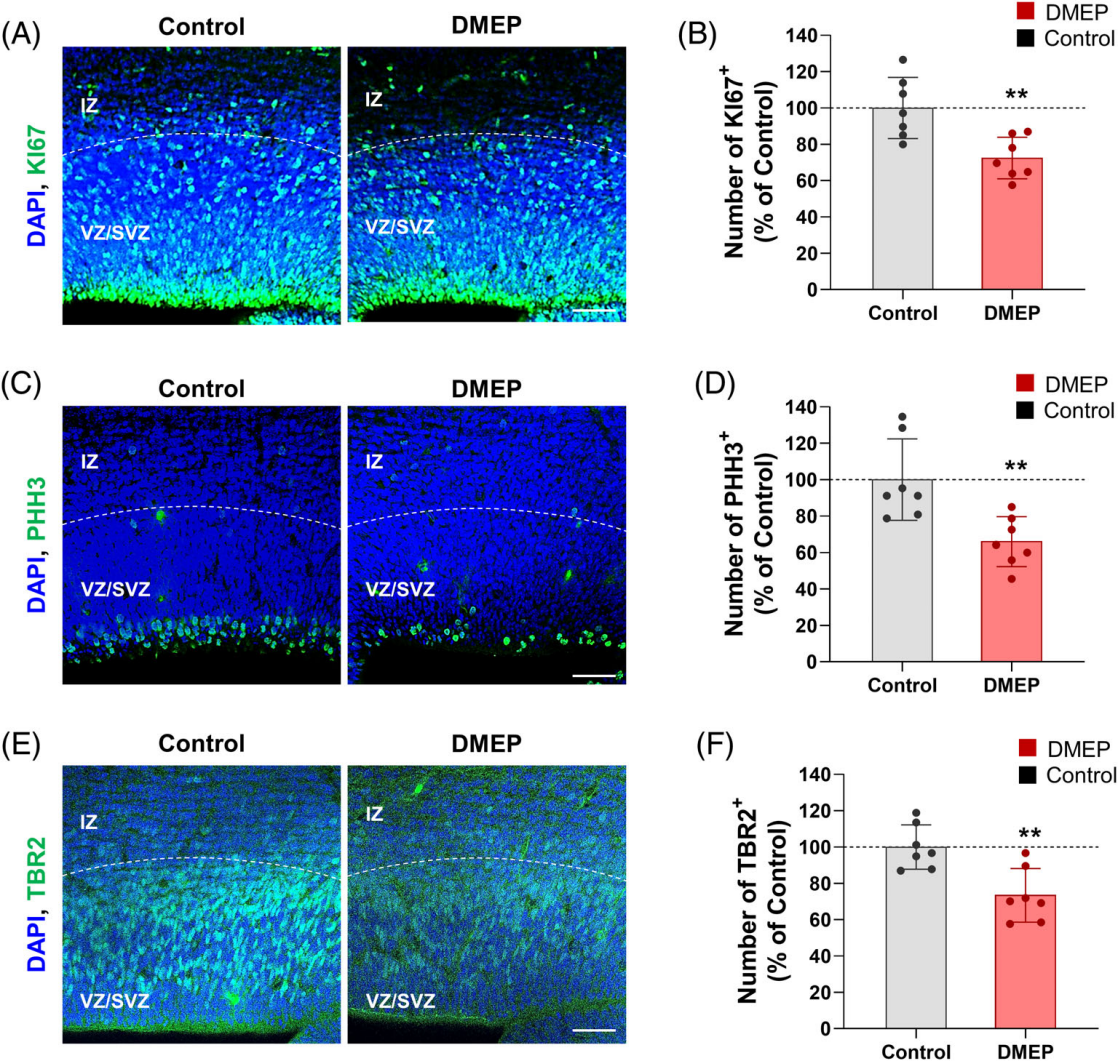
Prenatal DMEP exposure reduces proliferating cells during developing cortex. (A) The number of KI67‐positive actively proliferating cells was markedly reduced in prenatal DMEP‐exposed brain sections. E14.5 brain samples from controls and prenatal DMEP‐exposed mice were immunostained with an anti‐KI67 antibody. Scale bars: 50 μm. (B) Quantification of the number of KI67‐positive cells shown in (A). *N* = 7 mice for each condition. Statistical significance was determined by one‐way ANOVA with Bonferroni correction test. Data are shown as relative changes versus controls. **p < 0.01. (C) The number of phosphor‐Histone H3‐positive mitotic phase cells was markedly reduced in prenatal DMEP‐exposed brain sections. E14.5 brain samples from controls and prenatal DMEP‐exposed mice were immunostained with an anti‐phosphor‐Histone H3 antibody. Scale bars: 50 μm. (D) Quantification of the number of mitotic phase cells at VZ surface shown in (C). *N* = 7 mice for each condition. Statistical significance was determined by one‐way ANOVA with Bonferroni correction test. Data are shown as relative changes versus controls. **p < 0.01. (E) The number of TBR2‐positive intermediate progenitors was markedly reduced in prenatal DMEP‐exposed brain sections. E14.5 brain samples from controls and prenatal DMEP‐exposed mice were immunostained with an anti‐TBR2 antibody. Scale bars: 50 μm. (F) Quantification of the number of intermediate progenitors shown in (E). *N* = 7 mice for each condition. Statistical significance was determined by one‐way ANOVA with Bonferroni correction test. Data are shown as relative changes versus controls. **p < 0.01.

To determine the effect of DMEP on proliferation during cortical development, we performed immunostaining with antibodies against the whole cell cycle marker Ki67 and the mitosis marker phospho‐Histone H3 in E14.5 brain slices of control and prenatally DMEP‐exposed mice (Figure 1A–D). The number of actively proliferating cells immunoreactive to the Ki67 antibody was reduced by 28% in prenatally DMEP‐exposed mice compared with control mice (Figure 1A,B). The number of mitotic phase cells at VZ surface marked by phospho‐Histone H3 was reduced by 44% in the prenatally DMEP‐exposed mice compared with controls (Figure 1C,D). Next, we performed immunostaining with the intermediate progenitor marker Tbr2 in brain slices of control and prenatally DMEP‐exposed mice. We also observed that the number of Tbr2‐positive cells was reduced by 27% in the prenatally DMEP‐exposed mice compared with controls (Figure 1E,F). These findings indicate that prenatal DMEP exposure impairs progenitor proliferation during cortical development.

### 3.2 Prenatal DMEP exposure changes the gene expression profile during cortical development

**FIGURE 2:**
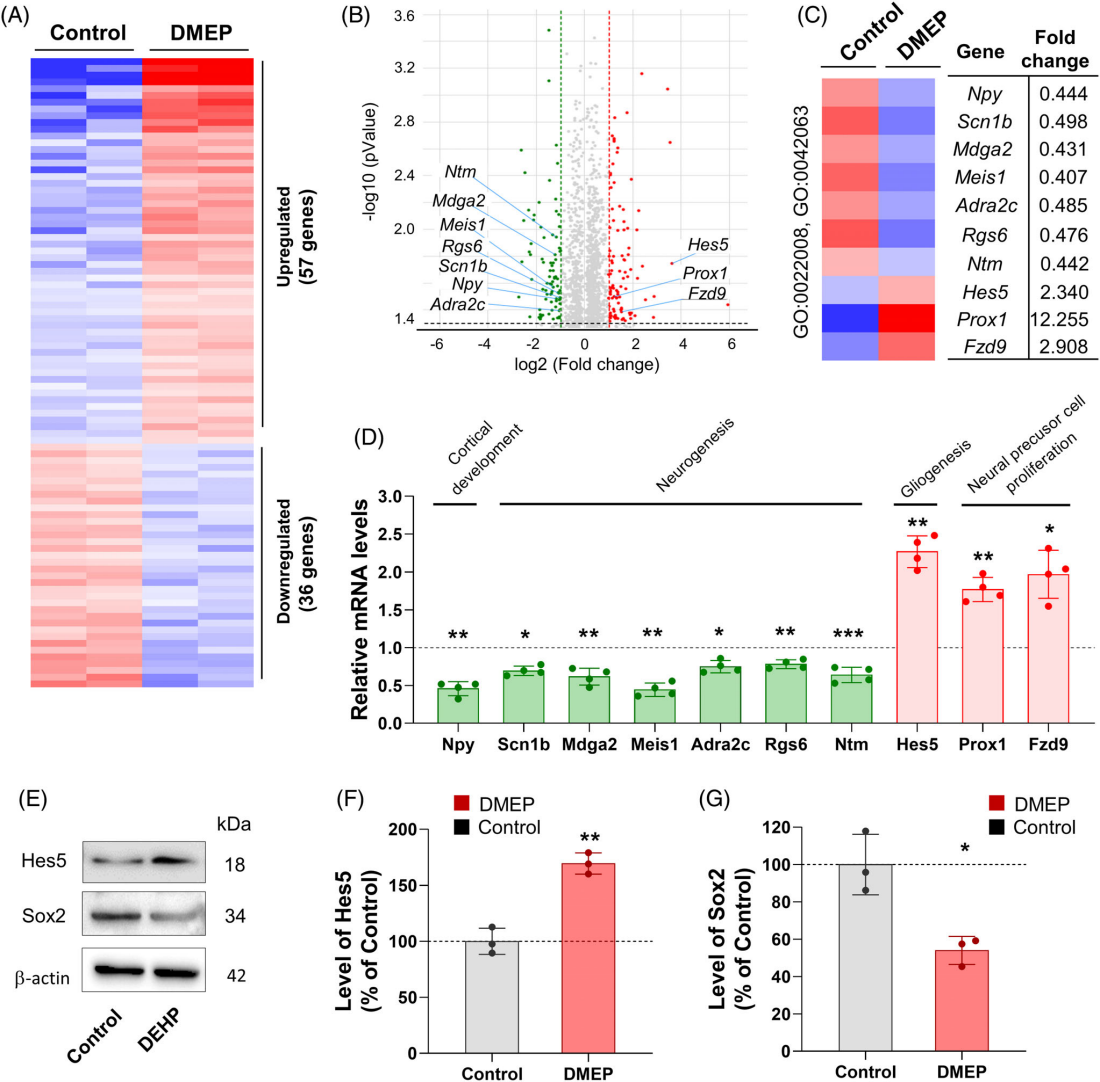
Prenatal DMEP exposure changes gene expression profile during developing cortex. (A) Heat map of RNA seq transcriptome analysis for different genes in E14.5 brain of control or prenatal DMEP exposed cortices (Fold change >2 or *p* < 0.05). (B) Volcano plot of DEGs for different genes in brain of control or prenatal DMEP exposed cortices. (C) The significantly enriched GO_BP terms for upregulated or downregulated genes by prenatal DMEP exposure. Annotated in GO (GO terms; neurogenesis [GO: 0022008], and gliogenesis [GO: 00042063]). (D) qPCR validated the upregulated or downregulated related to the GO terms. (E) The expression levels of Hes5 and Sox2 were detected by western blot in cortex of control or prenatal DMEP‐exposed cortices at E 14.5. (F, G) Quantification of western blots in (E). The protein levels were normalized to β‐action expression. *n* = 3. Data are presented as mean ± SEM. Statistical significance was determined by ANOVA with Bonferroni correction test. Data are shown as relative changes versus controls. *p < 0.05, **p < 0.01.

To determine the effects of DMEP on the mRNA transcriptome pattern during cortical development, we profiled global transcriptome expression by using the Quant‐Seq 3′ mRNA‐Seq Library Prep Kit FWD and NextSeq 500/550. Total RNA was acquired from the cortical tissue of E14.5 prenatally DMEP‐exposed mice and control mice. We found that 93 mRNAs were differentially expressed including 57 significantly upregulated genes and 36 significantly downregulated genes (Figure 2A). GO analysis revealed that the regulated genes were associated with cortical development, neurogenesis, and gliogenesis (Figure 2A–C). Next, we explored how DMEP exposure affected neurogenesis and gliogenesis at the molecular level during cortical development. Among the differentially regulated genes identified by global genome analysis, we found seven downregulated genes, *Nyp*, *Scn1b*, *Mdga2*, *Meis1*, *Adra2c*, *Rgs6*, and *Ntm*, that regulate neurogenesis (Figure 2B,C). We also found three upregulated genes, *Hes5*, *Prox1*, and *Fzd9*, that are related to gliogenesis and neuronal neural precursor cell proliferation (Figure 2B,C). To confirm the gene expression profiles in DMEP‐exposed cortices, we performed the mRNA expression levels of genes related to specific GO terms by real‐time PCR. First, we assessed the transcript levels of genes related to GO terms such as GO:0021987 (cortical development) and GO:0022008 (neurogenesis) in DMEP‐exposed cortices. The mRNA levels of the GO:0021987 (cortical development)‐related gene Npy were decreased by 0.46‐fold in DMEP‐exposed cortices compared with control cortices (Figure 2D). We also observed that the mRNA levels of genes related to GO:0022008 (neurogenesis), that is, *Scn1b*, *Mdga2*, *Meis1*, *Adra2c*, *Rgs6*, and *Ntm*, were decreased by 0.69‐, 0.79‐, 0.64‐, 0.62‐, 0.61‐ and 0.44‐fold, respectively, in DMEP‐exposed cortices compared with control cortices (Figure 2D). Next, we assessed the transcript levels of genes related to GO terms such as GO:0042063 (gliogenesis) and GO:2000177 (neural precursor cell proliferation) in DMEP‐exposed cortices. The mRNA levels of genes related to GO:2000177 (neural precursor cell proliferation), that is, *Prox1* and *Fzd9*, were increased by 1.77‐ and 1.97‐fold, respectively, in DMEP‐exposed cortices compared with control cortices (Figure 2D). Interestingly, the mRNA levels of the GO:0042063 (gliogenesis)‐related gene *Hes5* were increased by 2.27‐fold in DMEP‐exposed cortices compared with control cortices (Figure 2D).

We examined whether prenatal DMEP exposure causes neurogenesis and gliogenesis in the developing cortex. First, using immunoblotting, we measured the level of Hes5, a marker for gliogenesis, in cortical lysates from prenatally DMEP‐exposed mice and control mice. We observed that the level of Hes5 was increased significantly by 69% in prenatally DMEP‐exposed mice compared with controls (Figure 2E,F). Next, we performed immunoblotting on cortical lysates with an antibody against Sox2, a marker for multipotential neural stem cells. We observed that the level of Sox2 was decreased by 46% in prenatally DMEP‐exposed mice compared with controls (Figure 2E,G). Together, these findings indicate that prenatal DMEP exposure impairs neurogenesis and gliogenesis.

### 3.3 Prenatal DMEP exposure affects brain morphogenesis in early postnatal brain

**FIGURE 3:**
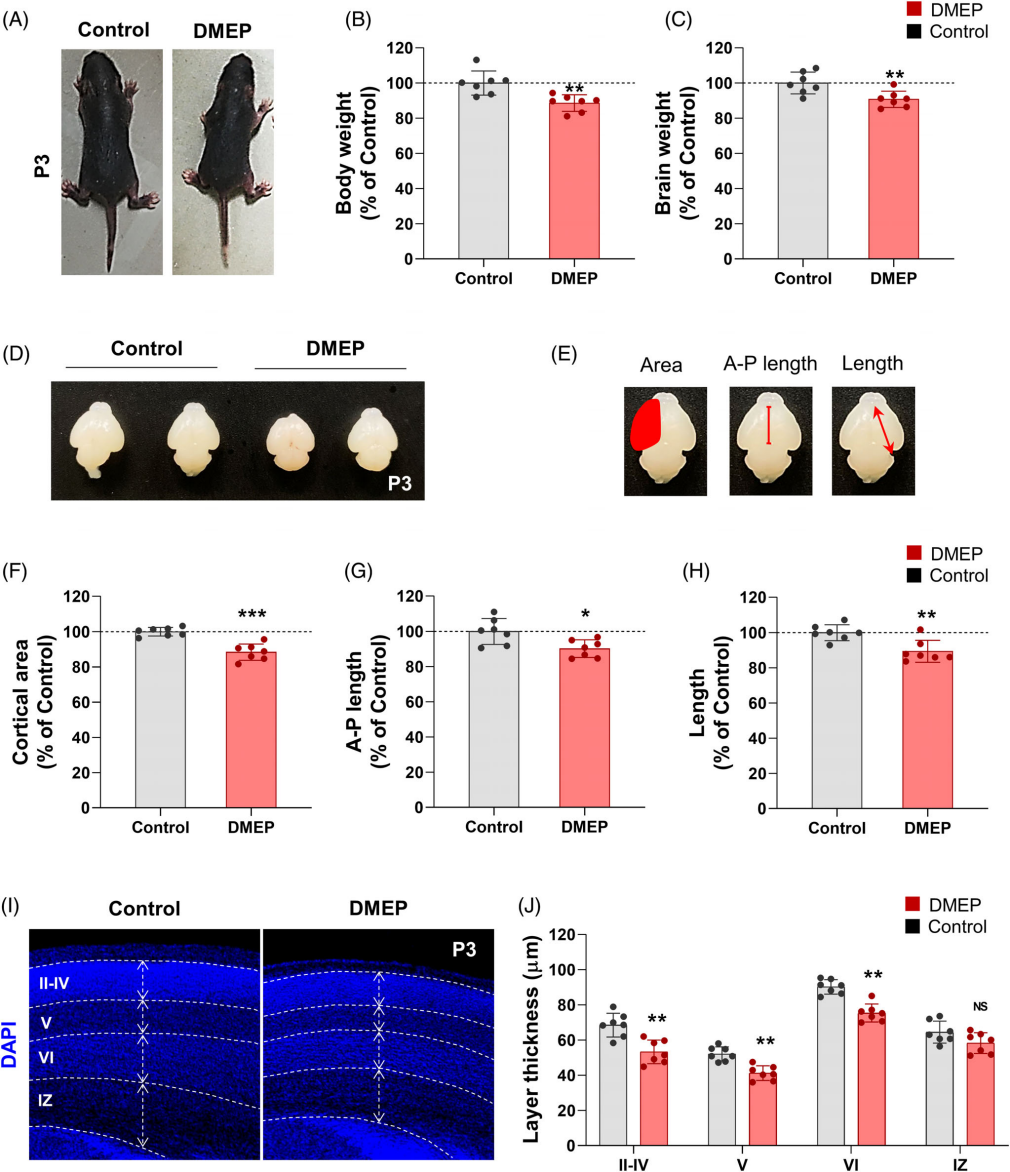
Prenatal DMEP exposure affects brain morphogenesis. (A) Representative image is body size in control and prenatal DMEP exposed mice at P3. The prenatal DMEP‐exposed mice were smaller than controls. (B, C) Body volume and length were reduced in prenatal DMEP‐exposed mice. *N* = 7 mice for each condition. Statistical significance was determined by one‐way ANOVA with Bonferroni correction test. Data are shown as relative changes versus controls. *p < 0.05, ***p < 0.001. (D) Brain size of prenatal DMEP‐exposed mice showed significantly smaller compared with the brain of control in E14.5 as well as P3. (E) Representative image is quantification of brain size at P3. (F, G) Cortical area, A‐P length and length were reduced in prenatal DMEP‐exposed brains. *N* = 7 mice for each condition. Statistical significance was determined by one‐way ANOVA with Bonferroni correction test. Data are shown as relative changes versus controls. *p < 0.05, **p < 0.01, ***p < 0.001. (I) Representative image is brain sections at P3 with DAPI staining. Scale bars: 200 μm. (J) Thickness of the cortical layer was reduced in prenatal DMEP‐exposed brains. *N* = 7 mice for each condition. Statistical significance was determined by one‐way ANOVA with Bonferroni correction test. Data are shown as relative changes versus controls. **p < 0.01.

To determine the effects of DMEP during brain development, we evaluated the body morphology of the prenatal DMEP‐exposed mice. Prenatal DMEP exposure slightly reduced body size in mice 3 days after birth (Figure 3A). The body weight and brain weight of prenatally DMEP‐exposed mice were reduced by 11% and 9%, respectively, compared with the control mice at the P3 stage (Figure 3B,C and Table 3). Next, we assessed the gross brain morphology of the prenatal DMEP‐exposed mice. We found that these mice displayed a slight reduction in brain size at P3 (Figure 3D). In addition, we found that prenatally exposed mice had a slight reduction in their cortical area, anteroposterior (A‐P) length, and cortical length compared with control mice at P3 (Figure 3E–H). The cortical volume, A‐P length, and cortical length of prenatally DMEP‐exposed mice were reduced by 15%, 11%, and 11%, respectively, compared with the control mice at the P3 stage (Figure 3E–H). Analysis of brain sections stained with DAPI revealed that the brains of prenatally DMEP‐exposed mice exhibited a thinner parietal cortex (Figure 3I). Next, we assessed the cortical layers of the prenatal DMEP‐exposed mice. All cortical layers were markedly reduced in the brains of prenatally DMEP‐exposed mice compared with control brains (Figure 1I,J). Interestingly, intermediate zone was no difference in the brains of prenatally DMEP‐exposed mice (Figure 3I,J). These findings indicate that prenatal DMEP exposure affects brain morphogenesis in the early postnatal brain.

**TABLE 3:** The body weight and brain weight at postnatal day 3 (P3).

ID	Control		ID	DMEP (50 mg/kg)	
	Body weight (mg)	Brain weight (mg)		Body weight (mg)	Brain weight (mg)
1	1210.5	126.7	8	1179.0	123.9
2	1486.4	150.8	9	1206.1	129.3
3	1316.2	137.4	10	1239.1	137.9
4	1333.4	148.1	11	1179.1	126.6
5	1331.4	142.2	12	1066.2	118.5
6	1229.2	132.2	13	1185.0	126.7
7	1290.3	135.4	14	1093.5	120.1

### 3.4 Prenatal DMEP exposure reduces neuron number in the parietal cortex

**FIGURE 4:**
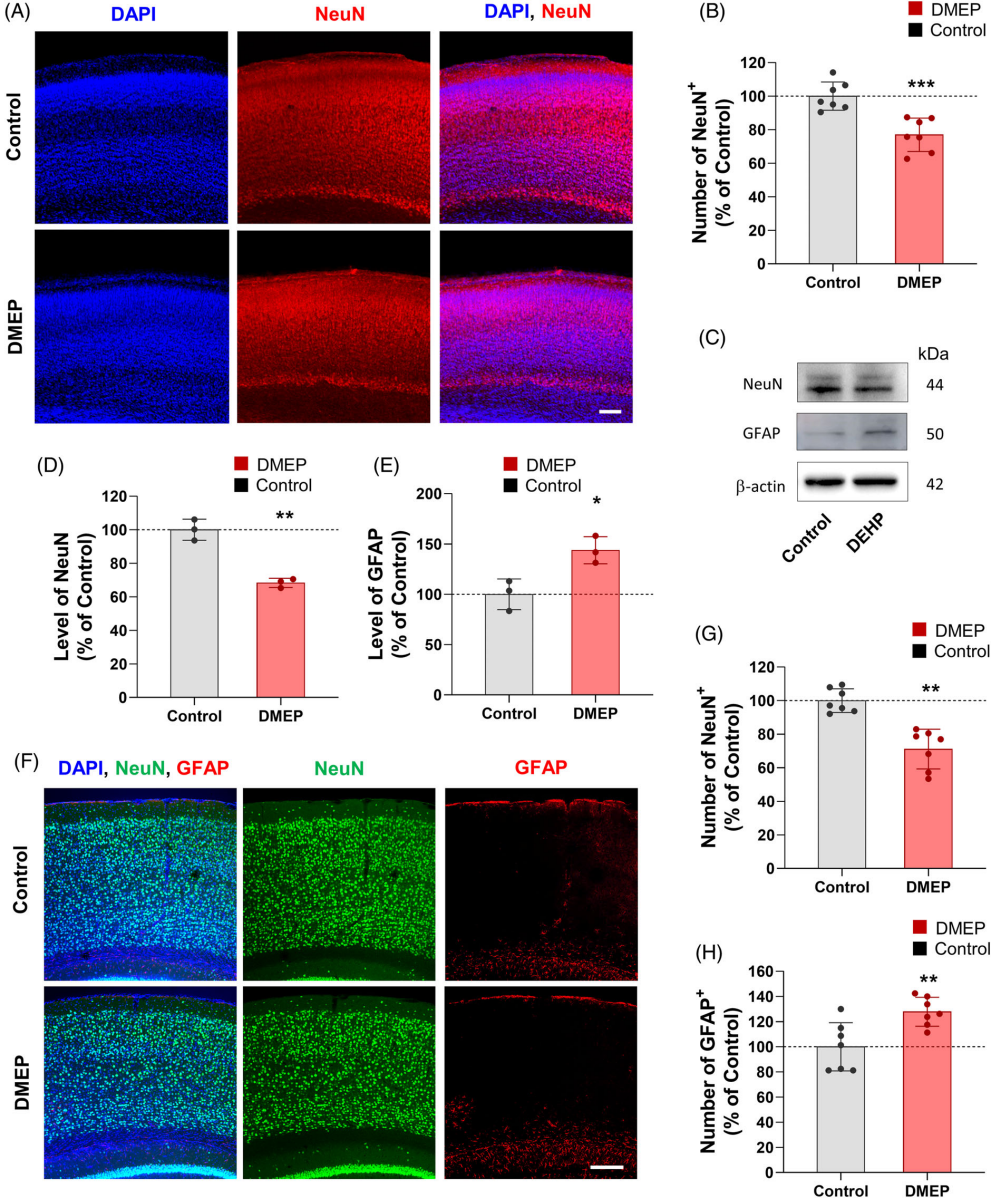
Prenatal DMEP exposure reduces neurons in the parietal cortex. (A) The number of NeuN‐positive neurons was markedly reduced in prenatal DMEP‐exposed brain sections. P3 brain samples from controls and prenatal DMEP‐exposed mice were immunostained with an anti‐NeuN antibody. Scale bars: 200 μm. (B) Quantification of the number of cortical neurons shown in (A). *N* = 7 mice for each condition. Statistical significance was determined by one‐way ANOVA with Bonferroni correction test. Data are shown as relative changes versus controls. ***p < 0.001. (C) The expression levels of NeuN and GFAP were detected by western blot in parietal cortex of control or prenatal DMEP‐exposed cortices at P3. (D, E) Quantification of western blots in (C). The protein levels were normalized to β‐action expression. *n* = 3. Data are presented as mean ± SEM. Statistical significance was determined by ANOVA with Bonferroni correction test. Data are shown as relative changes versus controls. *p < 0.05, **p < 0.01. (F) The number of NeuN‐positive neurons and GFAP‐positive astrocyte was markedly changed in prenatal DMEP‐exposed brain sections. P56 brain samples from controls and prenatal DMEP‐exposed mice were immunostained with anti‐NeuN or anti‐GFAP antibody. Scale bars: 200 μm. (G, H) Quantification of the number of cortical neurons and astrocytes shown in (F). *N* = 7 mice for each condition. Statistical significance was determined by one‐way ANOVA with Bonferroni correction test. Data are shown as relative changes versus controls. **p < 0.01.

The smaller brain morphology suggested that there was a reduction in neurons in the prenatally DMEP‐exposed cortices. To explore this possibility, we measured the neuron numbers in the postnatal parietal cortex. First, we used immunostaining with NeuN, a neural marker, to quantify the number of neurons in P3 prenatally DMEP‐exposed mice and control mice. We found that the number of NeuN‐positive cells was reduced by 23% in the cortices of prenatally DMEP‐exposed mice compared with control mice (Figure 4A,B). Next, we assessed NeuN and GFAP in cortical lysates at P3 using immunoblotting (Figure 4C). We observed that the level of NeuN was decreased by 32% in prenatally DMEP‐exposed mice compared with controls (Figure 4C,D). However, the level of GFAP was increased by 44% in prenatally DMEP‐exposed mice compared with control mice (Figure 4C,E).

Next, to confirm whether prenatally DMEP‐exposed mice exhibit altered neuron numbers and positioning in the cortices, DMEP (50 mg/kg) was administered to pregnant mice orally once a day until breastfeeding of the fetus was stopped. Fifty‐six days after birth, the neurons and glial cells of prenatally DMEP‐exposed mice and control mice were assessed by immunostaining. Compared with the control cortices, prenatally DMEP‐exposed cortices showed a 25% reduction in the number of NeuN‐positive neurons at the P56 stage (Figure 4F,G). Meanwhile, the number of GFAP‐positive astrocytes was increased by 33% in the prenatally DMEP‐exposed cortices compared with the control cortices (Figure 4F,H). These findings indicate that prenatal DMEP exposure can lead to abnormal neurogenesis and astrogenesis in brain development.

### 3.5 DMEP exposure disrupts dendritic spine architecture and synaptic activity in cortical neurons

**FIGURE 5:**
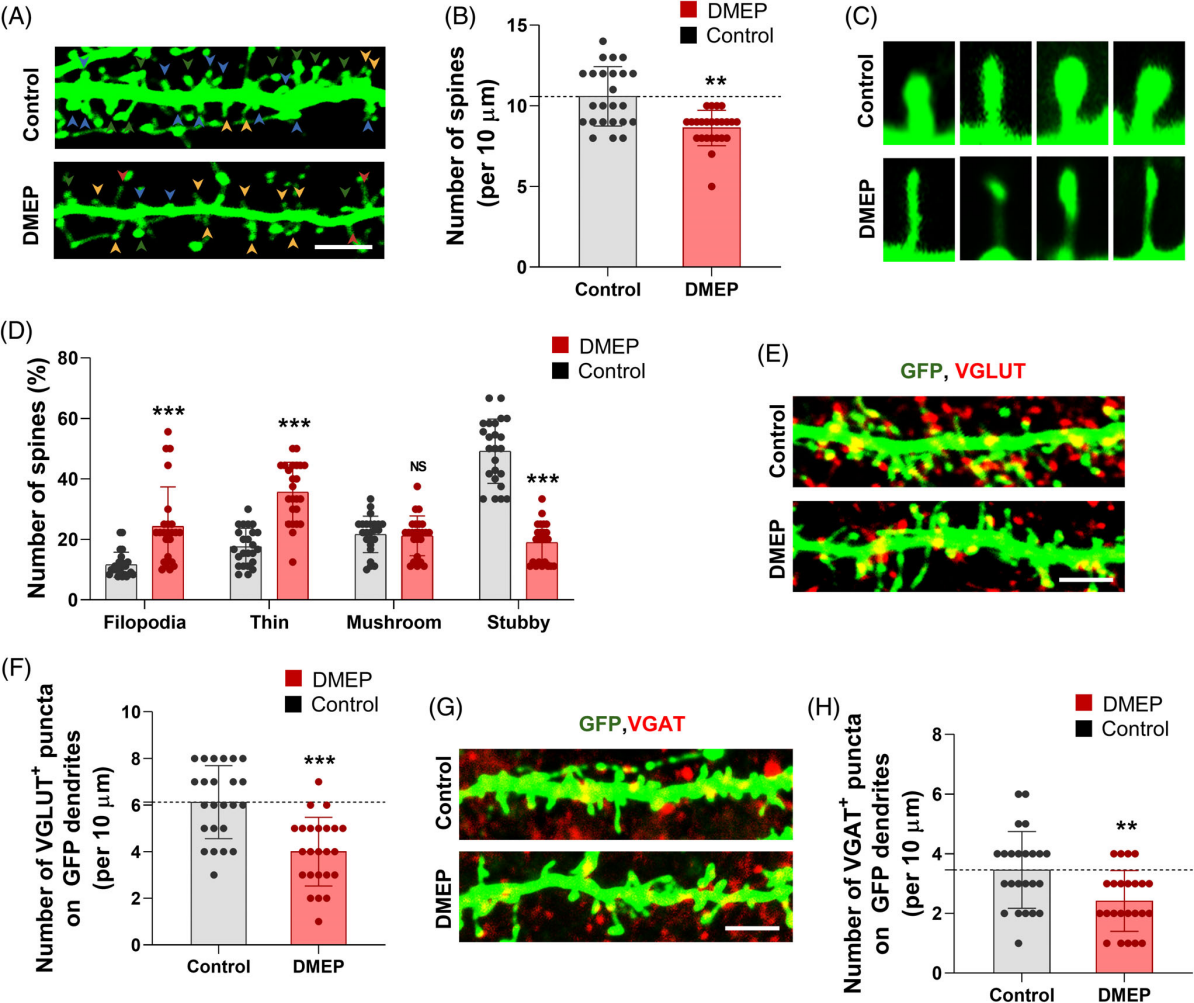
DMEP exposure affects synaptic formation and Function in cultured neurons. (A) DMEP modifies dendrites spine density in cultured cortical neurons. Cultured cortical neurons were isolated from E14.5 embryos, cultured, and transfected with a GFP plasmid at DIV 10. After 4 days, neurons were treated with 100 μM DMEP for 24 h. Scale bars: 5 μm. (B) Quantification of the number of dendritic spines in each condition. *n* = 24 neurons from three independent cultures using three mice for each condition. Statistical significance was determined by one‐way ANOVA with Bonferroni correction test. Data are shown as relative changes versus controls. **p < 0.01. (C) Higher magnification images of dendritic spines. Exposure of DMEP leads to abnormal dendritic spine morphology. (D) DMEP remodels the composition of dendritic spine types in cultured cortical neurons. *n* = 12 cultured cortical neurons and 300 dendritic spines for each condition. Statistical significance was determined by two‐way ANOVA with Bonferroni correction test. Data are shown as relative changes versus controls. ***p < 0.001. (E) Excitatory synapses were assessed by immunostaining using a VGLUT antibody. Scale bars: 5 μm. (F) Quantification of the number of excitatory synapses shown in (E). *n* = 24 neurons from three independent cultures using three mice for each condition. Statistical significance was determined by one‐way ANOVA with Bonferroni correction test. Data are shown as relative changes versus controls. ***p < 0.001. (G) Inhibitory synapses were assessed by immunostaining using a VGAT antibody. Scale bars: 5 μm. (H) Quantification of the number of excitatory synapses shown in (G). *n* = 24 neurons from three independent cultures using three mice for each condition. Statistical significance was determined by one‐way ANOVA with Bonferroni correction test. Data are shown as relative changes versus controls. **p < 0.01.

To determine the effects of DMEP on dendritic spine architecture in cortical neurons, we transfected into cortical neurons with plasmids encoding GFP at day in vitro 10 (DIV 10). Then, transfected cortical neurons were exposed to 100 μM DMEP for 24 h at DIV14. We assessed the dendritic spines by GFP immunostaining in cortical neurons. We observed that the dendritic spine density was reduced by 19% in DMEP‐exposed neurons compared with control neurons (Figure 5A,B). Compared with control neurons, DMEP‐exposed neurons displayed marked decreases in stubby spines (Figure 5A,C,D). We observed that the stubby spine density was reduced by 61% in DMEP‐exposed neurons compared to controls (Figure 5A,D). Meanwhile, compared with control neurons, DMEP‐exposed neurons displayed marked increases in filopodia and thin spines (Figure 5A,C,D). The filopodia and thin spine density were increased by 108% and 103%, respectively, in DMEP‐exposed neurons compared with control neurons (Figure 5A,D). Next, we determine whether DMEP changes the excitatory and inhibitory synapses by immunostaining with antibodies to synaptic markers VGLUT (excitatory) and VGAT (inhibitory). We observed that the numbers of excitatory and inhibitory synapses were reduced by 35% and 40%, respectively, in DMEP‐exposed neurons compared with control neurons (Figure 5E–H). Taken together, these findings indicate that DMEP exposure impairs the dendritic spine architecture and synapses in cortical neurons.

**FIGURE 6:**
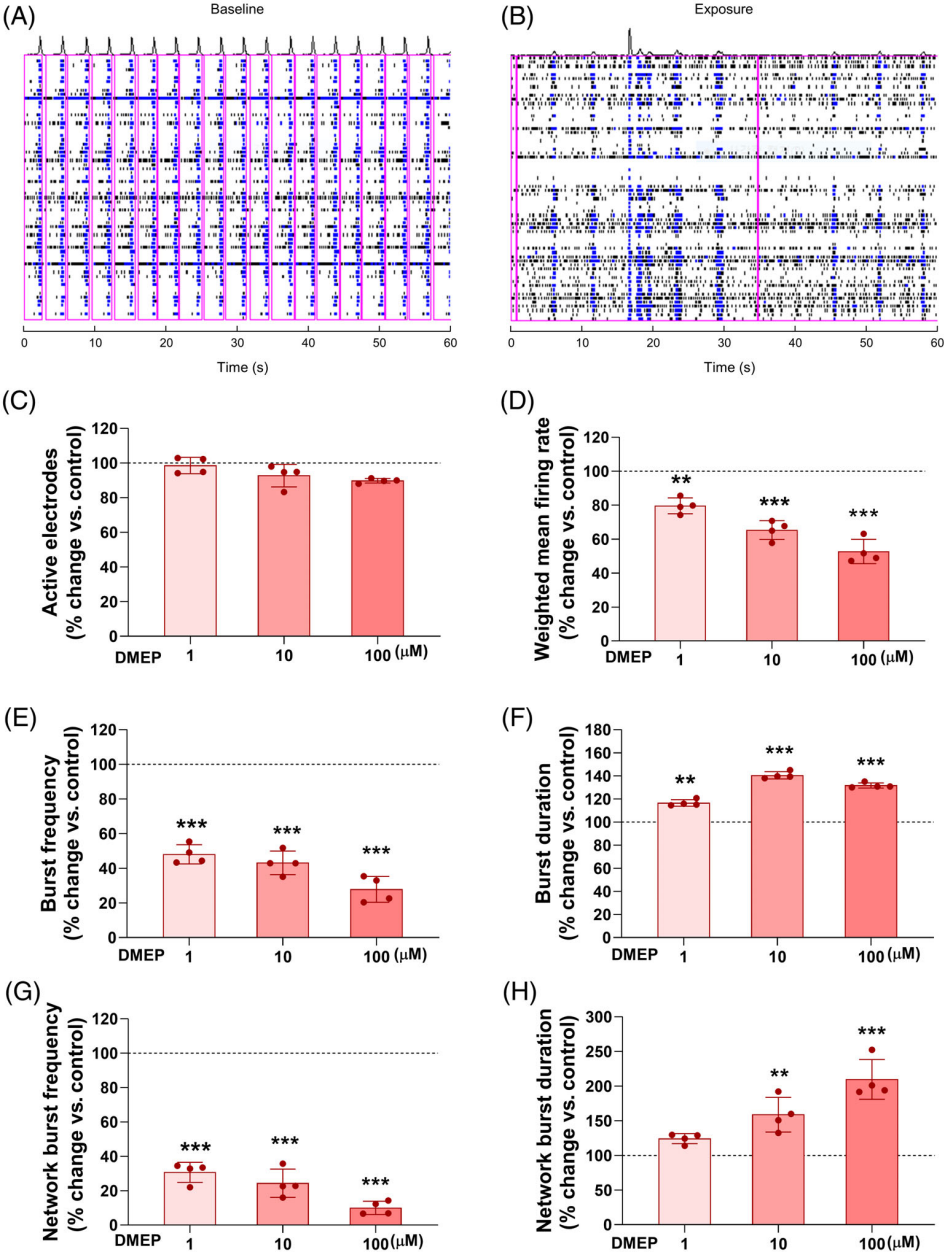
DMEP exposure affects neural activity in cultured neurons. Spike raster plots illustrating the pattern of a representative well of baseline (A) and the same well following exposure (right) to 100 μM DMEP (B). Each row depicts one electrode in a well and spikes are depicted in black, and bursts are depicted in blue. Cortical neurons were cultured for 14 days, and then 100 μM DMEP was added for 24 h. *n* = 5. Effects on active electrodes (C), weighed mean firing rate (D), burst frequency (E), burst duration (F), network burst frequency (G), and network burst duration (H) are depicted as average in % change of control (0.1% DMSO control set to 100%; dashed line). Data are presented as mean ± SEM. Statistical significance was determined by ANOVA with Bonferroni correction test. Data are shown as relative changes versus controls. **p < 0.01, ***p < 0.001.

The abnormal dendritic spine number and morphology suggested that there is synapse dysfunction in cortical neurons. Next, to determine the effects of DMEP on functional relevance of abnormal neural activity, we cultured cortical neurons on a multiwell MEA plate for 14 days and assessed neural activity and network activity in the presence or absence of DMEP. We observed that the spiking and bursting activity combined with network burst duration were reduced by acute DMEP exposure (Figure 6A,B). The activity recorded by electrodes was not significantly different between acute DMEP‐exposed neurons and controls (Figure 6A–C). Interestingly, the weighted mean firing rate and burst frequency were reduced by acute DMEP exposure (Figure 6D,E). However, the burst duration was increased by acute DMEP exposure (Figure 6F). Next, we measured the network activity in the presence or absence of DMEP. We observed that the network burst frequency was reduced in a dose‐dependent manner by acute DMEP exposure (Figure 6G). Otherwise, the network burst duration was increased in a dose‐related manner by acute DMEP exposure (Figure 6H). Together, these findings indicate that acute DMEP exposure impairs neural activity and network activity in cultured cortical neurons.

### 3.6 Prenatal DMEP exposure disrupts architecture and synapse formation in the parietal cortex

**FIGURE 7:**
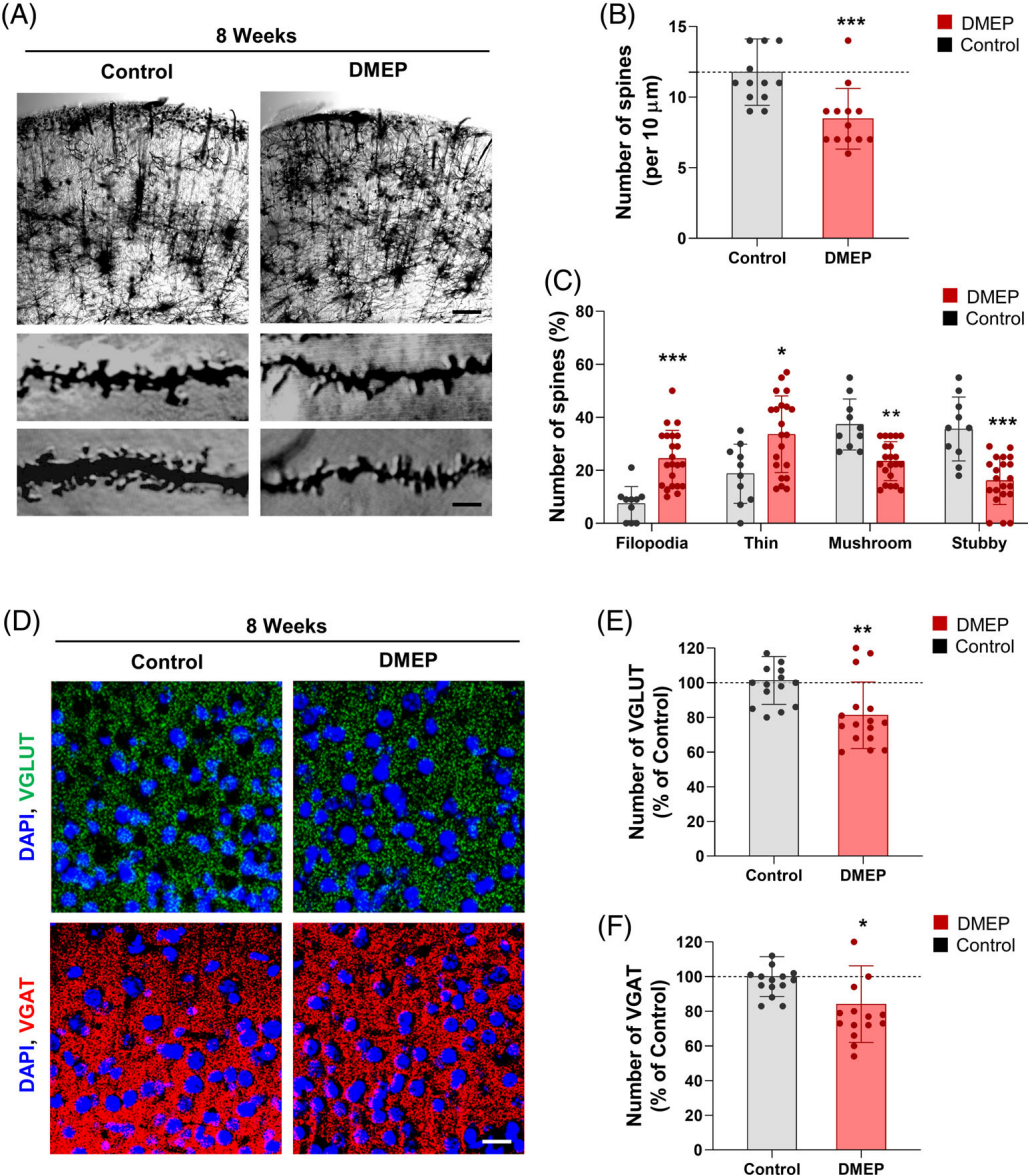
Prenatal DMEP exposure affects dendritic spine formation in the parietal cortex. (A) Dendritic spines were assessed in cortical tissues from mice exposed with either DMEP (50 mg/kg) or saline (control) orally once a day for 8 weeks. Golgi staining showed a decrease in dendritic spines in DMEP‐exposed cortices. Scale bar: 200 μm (top panels) and 5 μm. (bottom panels). (B) Quantification of the number of dendritic spines in each condition. *n* = 12 neurons from 3 mice for each condition. Statistical significance was determined by two‐way ANOVA with Bonferroni correction test. Data are shown as relative changes versus controls. ***p < 0.001. (C) DMEP remodels the composition of dendritic spine types in cortices. *n* = 10 sections from 3 mice for each condition. Statistical significance was determined by two‐way ANOVA with Bonferroni correction test. Data are shown as relative changes versus controls. *p < 0.05, **p < 0.01, ***p < 0.001. (D) Excitatory and inhibitory synapses were assessed by immunostaining with a VGLUT and VGAT antibody. Scale bar, 10 μm. (E), (F) Quantification of the number of synaptic puncta in each condition. *n* = 15 sections from 3 mice for each condition. Statistical significance was determined by two‐way ANOVA with Bonferroni correction test. Data are shown as relative changes versus controls. *p < 0.05, **p < 0.01.

To determine the effects of DMEP on dendritic spine and synapse formation in the parietal cortex, we assessed dendrite morphogenesis by Golgi staining in P56 brain slices of control and prenatally DMEP‐exposed mice. We observed that the total dendritic spine density was decreased by 28% in prenatally DMEP‐exposed cortices compared with controls (Figure 7A,B). We also observed that prenatally DMEP‐exposed cortices displayed marked decreases in the numbers of mushroom head and stubby spines compared with controls. However, the number of filopodia and thin spines was markedly increased in prenatal DMEP‐exposed cortices compared with controls (Figure 7A). Based on quantitative analysis, the mushroom head and stubby spine density were reduced by 37% and 65%, respectively, in prenatal DMEP‐exposed cortices compared with control cortices (Figure 7A,C). In contrast, the filopodia and thin spine density were increased by 230% and 80%, respectively, in DMEP‐exposed cortices compared with controls (Figure 7A,C). These results indicate that prenatal DMEP exposure affects dendrite architecture in the developing parietal cortex.

Next, we assessed the excitatory synapse density in the parietal cortex of prenatally DMEP‐exposed mice or controls using immunostaining with a VGLUT antibody. The VGLUT puncta was reduced by 20% in prenatally DMEP‐exposed cortices compared with controls (Figure 7D,E). We also assessed the inhibitory synapse density in the parietal cortex of prenatally DMEP‐exposed mice or control mice using immunostaining with a VGAT antibody. Prenatally DMEP‐exposed cortices displayed a 16% reduction in the VGAT puncta compared with control cortices (Figure 7D,F). Taken together, these results indicate that prenatal DMEP exposure leads to synaptic dysfunction in the parietal cortex by affecting dendritic spine and synaptic formations.

### 3.7 Prenatal DMEP exposure changes the gene expression profile in the parietal cortex

**FIGURE 8:**
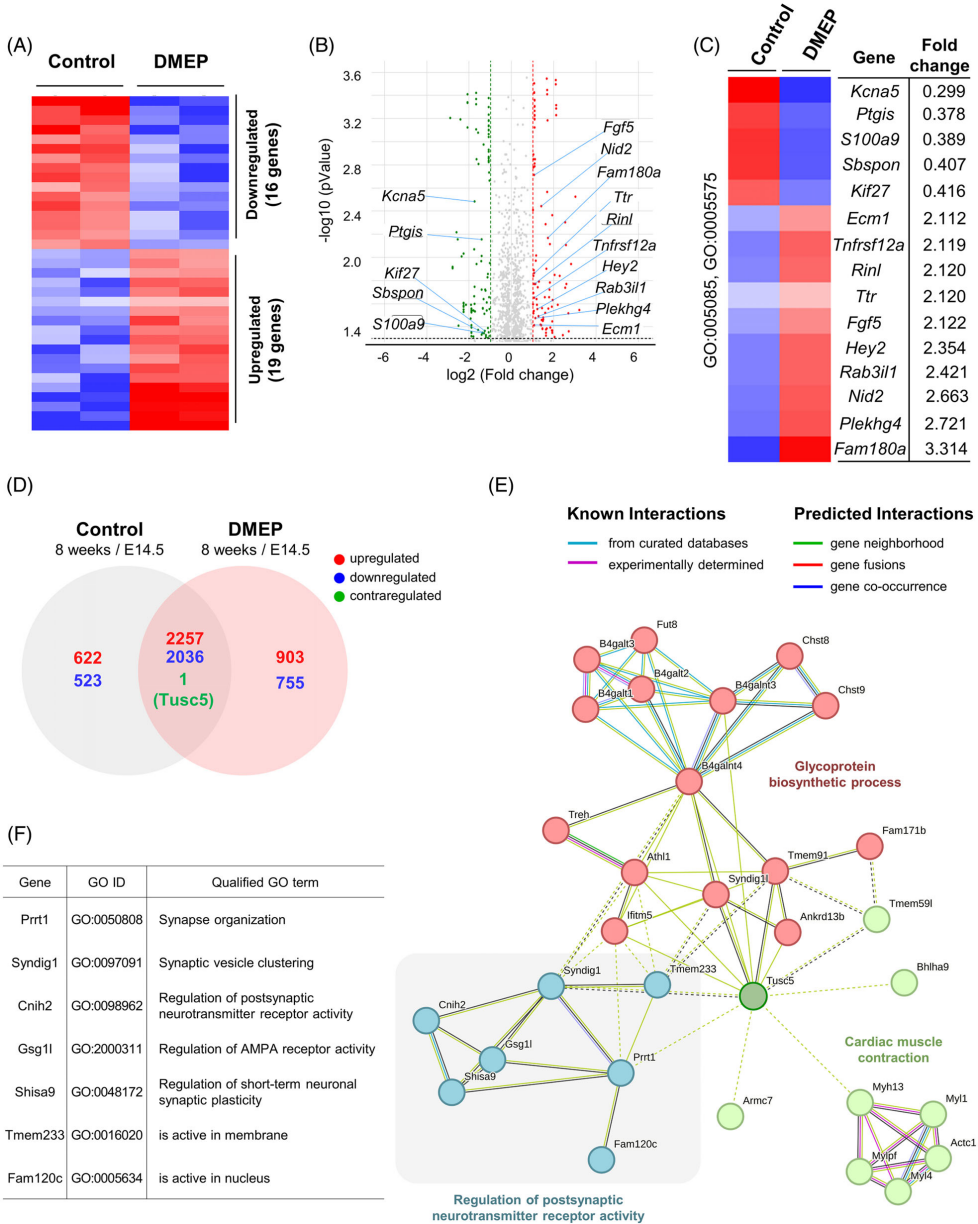
Prenatal DMEP exposure changes gene expression profile in parietal cortex. (A) Heat map of RNA seq transcriptome analysis for different genes in 8 weeks brain of control or prenatal DMEP exposed cortices (Fold change >2 or *p* < 0.05). (B) Volcano plot of DEGs for different genes in brain of control or prenatal DMEP exposed cortices. (C) The significantly enriched GO_BP terms for upregulated or downregulated genes by prenatal DMEP exposure. Annotated in GO (GO terms; extracellular region [GO: 0005576], guanyl‐nucleotide exchange factor activity [GO: 0005085], and protein binding [GO: 0005515]). (D) Venn diagram of DEGs in the cortices between controls (8 weeks/E14.5) and prenatal DMEP‐exposed brains (8 weeks/E14.5). (E) Protein interaction network of Tus5 was created using STRING. (F) The enriched GO terms for interacting proteins of Tus5.

To determine the effects of DMEP on the mRNA transcriptome pattern in the parietal cortex, we profiled the gene expression of the global transcriptome. Total RNA was acquired from the cortical tissue of P56 prenatally DMEP‐exposed mice and control mice. We found that 35 mRNAs were differentially expressed including 19 upregulated genes and 16 markedly downregulated genes (Figure 8A). GO analysis revealed that regulated genes were associated with dendritic spine formation, the extracellular region, and protein binding (Figure 8A–C). We explored how prenatal DMEP exposure affects dendritic spine formation and neural activity in the parietal cortex at the molecular level. Among the differentially regulated genes identified by global genome analysis, we found differentially regulated genes such as *Fgf5*, *Sbspon*, *Ecm1*, *Ttr*, *Fam180a*, *Nid2*, *S100a9*, and *Kif27* that are related to GO:0005575 (extracellular region) (Figure 8B,C). Moreover, we also found differentially regulated genes such as *Plekhg4*, *Rinl*, and *Rab3il1* that are related to dendritic spine formation GO:0005085 (guanyl‐nucleotide exchange factor activity) (Figure 8B,C). Next, we determined whether DMEP affects the change of gene expression patterns in the developing parietal cortex and adult parietal cortex. For this, we profiled global transcriptome expression in the E14.5 and P56 cortices (Figure 8D). In P56 control cortices, we found that 5439 mRNAs were differentially expressed including 2879 upregulated genes and 2560 downregulated genes compared with E14.5 control cortices (Figure 8D). In P56 prenatally DMEP‐exposed cortices, we found that 5952 mRNAs were differentially expressed including 3161 upregulated and 2791 downregulated genes compared with E14.5 prenatally DMEP‐exposed cortices (Figure 8D). Interestingly, we found that the *Tusc5* gene is dysregulated in prenatally DMEP‐exposed cortices compared with control cortices. The transcription level of *Tusc5* was reduced in P56 control cortices compared with E14.5 control cortices. However, the transcription level of *Tusc5* was increased in P56 prenatally DMEP‐exposed cortices compared with E14.5 prenatally DMEP‐exposed cortices (Figure 8D). The search tool for retrieval of interacting genes (STRING) analysis revealed that the *Tusc5* gene interacted with proteins such as Prrt1, Syndig1, Cnih2, Gsg1l, Shisa9, Tmem233, and Fam120c, which regulate postsynaptic neurotransmitter receptor activity (Figure 8E,F). Together, these results indicate that prenatal DMEP exposure impairs synaptic formation and function by regulating the mRNA transcriptome pattern in mice.

### 3.8 Prenatal DMEP exposure results in abnormal behaviors

**FIGURE 9:**
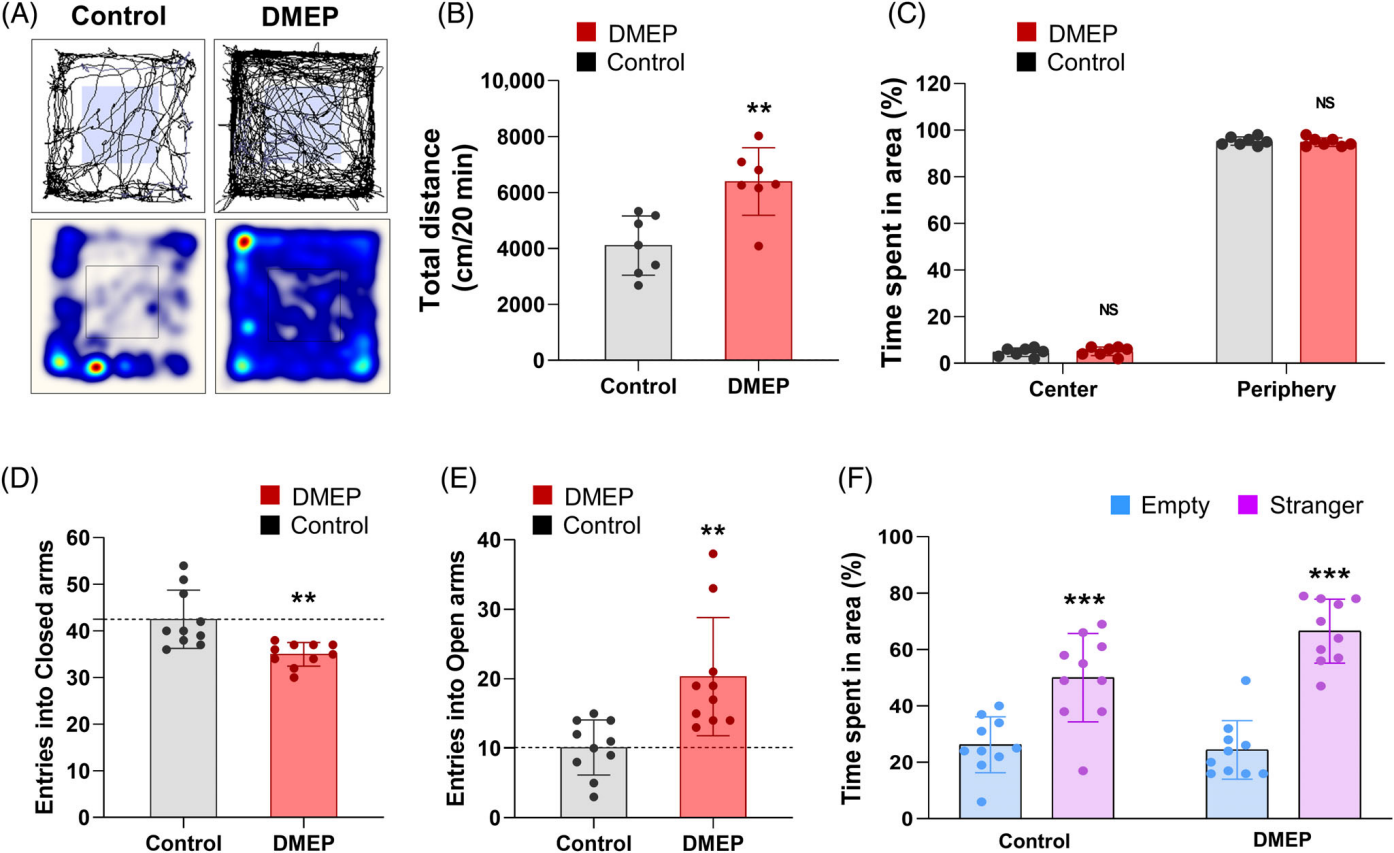
Prenatal DMEP exposure shows abnormal behaviors. (A) Representative image is locomotor activity in control and prenatal DMEP‐exposed mice at 8 weeks. (B, C) Motor activities were assessed by measuring the total distance of movement during the open field test. *N* = 7 mice for controls and prenatal DMEP‐exposed mice. Total time spent in the center and periphery was quantified in the open field test. *N* = 7 mice for controls and prenatal DMEP‐exposed mice. Statistical significance was determined by one‐way ANOVA with Bonferroni correction test. Data are shown as relative changes versus controls. **p < 0.01. (D, E) Total entries in open arms or closed arms were quantified in the EPM test. *n =* 10 mice for each condition. Statistical significance was determined by one‐way ANOVA with Bonferroni correction test. Data are shown as relative changes versus controls. **p < 0.01. (F) The three‐chamber social test assessed social behaviors in control and prenatal DMEP‐exposed mice. *N* = 10 mice for controls and prenatal DMEP‐exposed mice. Statistical significance was determined by one‐way ANOVA with Bonferroni correction test. ***p < 0.001 versus empty or stranger condition.

To determine whether prenatal DMEP exposure affects behavioral outcomes, DMEP (50 mg/kg) was administered to pregnant mice once a day from E0 to the cessation of breastfeeding the fetus. Eight weeks after birth, we performed several behavioral tests on prenatally DMEP‐exposed mice or control mice. First, we use the locomotor activity test to measure the activity and stereotyped behavior of mice in a novel environment. The locomotor activity was increased in prenatally DMEP‐exposed mice, as their total travel distance 56% higher than the distance traversed by controls (Figure 9A,B). We also measured the anxiety levels of prenatally DMEP‐exposed mice and controls by measuring their time spent in the center area of the locomotor activity device. We found no significant difference in the time spent in the center area of the locomotor activity device between prenatally DMEP‐exposed mice and control mice (Figure 9A,C). Next, we confirmed anxiety behaviors by the EPM test. We observed that the time spent in the closed arm was reduced by 18% in prenatally DMEP‐exposed mice compared with controls (Figure 9D). Meanwhile, the prenatally DMEP‐exposed mice spent 101% more time in the open arm than the control mice (Figure 9E). Finally, we assessed sociability in prenatally exposed mice and controls using a three‐chamber test. We found no significant difference in the proportion of time spent in the area with a stranger rather than an empty area by prenatal DMEP‐exposed mice and control mice (Figure 9F). Taken together, these results indicate that prenatal DMEP exposure is associated with a variety of neurodevelopmental symptoms, including hyperactivity and reduced anxiety behaviors.

## 4 DISCUSSION

**FIGURE 10:**
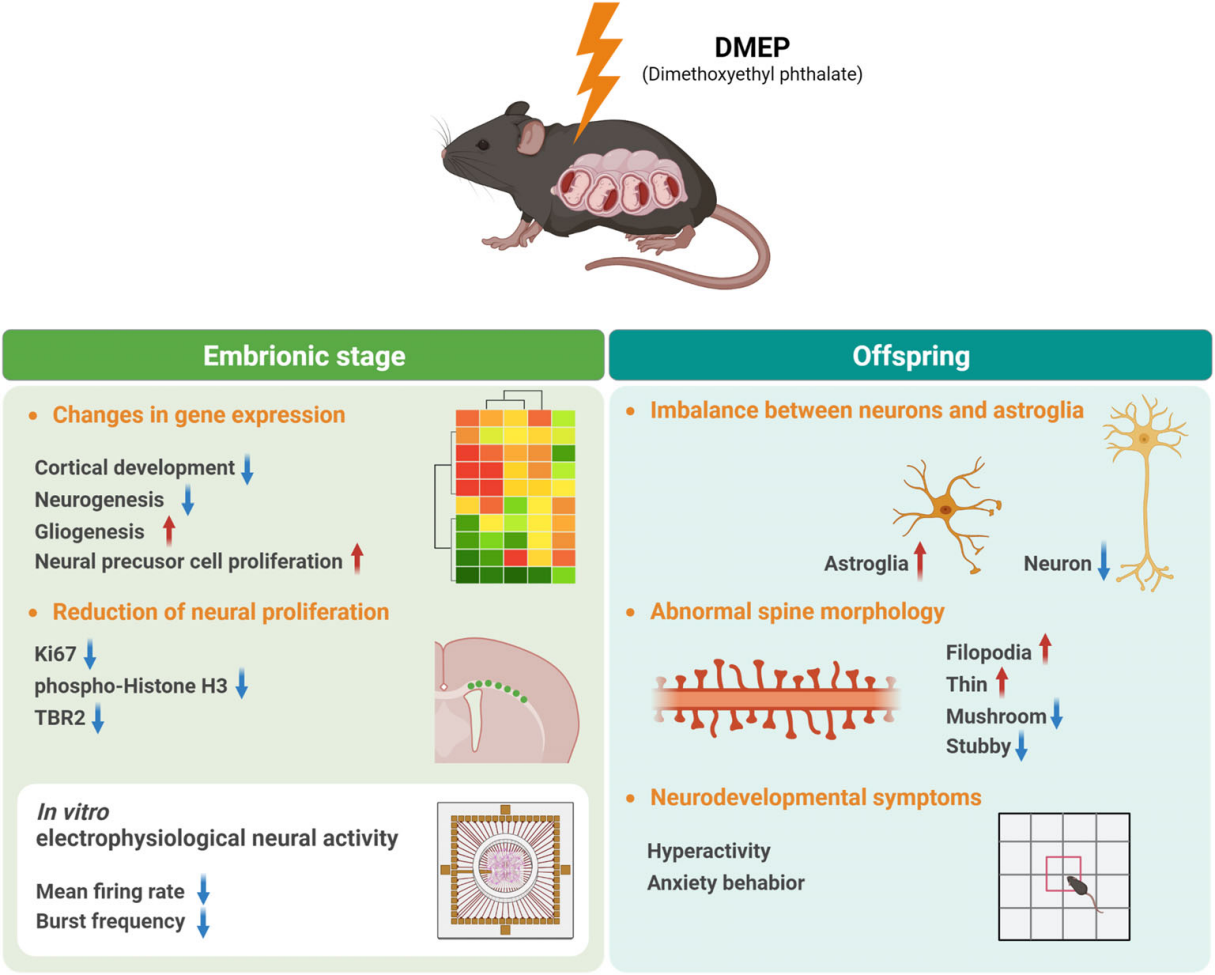
Schematic model illustrating an effect of prenatal DMEP exposure on cortical development and synaptic function in the mice.

Recent epidemiological investigation has reported that exposure to phthalates during pregnancy leads to offspring with abnormal brain function including ADHD and intellectual disability. Here, we provide evidence that prenatal DMEP exposure causes abnormal behaviors by impairing brain morphogenesis and synaptic activity in mice (Figure 10). Our work provides insights into the molecular pathogenesis of phthalates‐related neurodevelopmental disorders.

We show that prenatal DMEP exposure resulted in a reduction in neuron number of the parietal cortex by inhibiting the proliferation. In a zebrafish study, phthalates exposure inhibits neurogenesis by downregulating estrogen receptor expression [35]. Additionally, our findings were consistent with previous findings that prenatal phthalate mixture exposure resulted in a reduction in neuron number of the medial prefrontal cortex (mPFC) in rats [36]. Specifically, DBP exposure alters neural proliferation and hippocampal neurogenesis in adult mice [19]. Moreover, postnatal DEHP exposure impairs neural progenitor cell proliferation and differentiation in the hippocampus of mice [37]. We also found that prenatal DMEP exposure changed the gene expression profile in the developing cortex. The cortical development‐ and neurogenesis‐related genes *Nyp*, *Scn1b*, *Mdga2*, *Meis1*, *Adra2c*, *Rgs6*, and *Ntm* were downregulated during cortical development in prenatally DMEP‐exposed cortices. In contrast, the neural precursor cell proliferation‐ and gliogenesis‐related genes *Prox1*, *Fzd9*, and *Hes5* were upregulated during cortical development in prenatally DMEP‐exposed cortices. We found that prenatal DMEP exposure increases the GFAP‐positive astrocytes in the offspring brain. Furthermore, we also found that prenatal DMEP exposure increased the expression of Hes5 in E14.5 cortical lysates. Previous study has shown that Hes5 promoted gliogenesis and inhibited generation of neurons [38]. Interestingly, Hes5 regulates the transition of neurogenesis and gliogenesis during cortical development [39]. The nuclear factor 1A (NF1A) is essential for Hes5 expression that activates gliogenesis in the developing brain [40]. Overall, these results suggest that prenatal DMEP exposure causes unbalanced neurogenesis and gliogenesis via upregulation of *Hes5*.

Abnormal dendrite architecture could contribute to neurodevelopmental disorders such as schizophrenia, Rett's syndrome and autism spectrum disorder, and ADHD [41, 42, 43, 44]. We found that prenatal DMEP exposure resulted in reduced numbers of dendritic spines and altered dendritic spine morphogenesis in the parietal cortex. Furthermore, prenatally DMEP‐exposed brains showed abnormal synaptic formation that is important for normal brain functions. Consistently, perinatal phthalates exposure impairs cognitive flexibility by reduction of neuron number, synapse number, and size in the mPFC [36]. We also found that acute DMEP exposure resulted in abnormal neural activity and neural networks in cortical neurons. Similarly, DEHP exposure modulates minisynaptic transmission of projection neurons by inhibiting the calcium channel in the *Drosophila* [45]. In addition, prenatal DEHP exposure impairs hippocampal synaptic activity by inhibiting dendritic spine formation and synaptic structure in male offspring [46]. We found that prenatal DMEP exposure changed postsynaptic neurotransmitter receptor activity in the parietal cortex and that prenatal DMEP exposure resulted in abnormal behaviors such as hyperactivity and reduced anxiety in the offspring. Previous studies have consistently shown that endocrine disruptors, bisphenols, and phthalates cause hyperactive behaviors in the rats [47]. More importantly, epidemiological evidences have proposed that prenatal phthalates exposure has been linked with the neurodevelopmental disorders such as ADHD and autism [48, 49]. Our study reveals that prenatal DMEP impairs dendrite architecture in cortical neurons and is critical in the pathogenesis of synaptic transmission‐associated neurodevelopmental disorders.

## AUTHOR CONTRIBUTIONS

Minhan Ka and Sung‐Ae Hyun conceived and supervised this study. Minhan Ka, Sung‐Ae Hyun, Moon Yi Ko, Heejin Park and Sun‐Hwa Chon designed the research and analyzed the data. Moon Yi Ko and Sun‐Hwa Chon performed the research. Minhan Ka, Sung‐Ae Hyun wrote the manuscript. Byoung‐Seok Lee and Sun‐Hwa Chon provided critical review of the manuscript. All authors reviewed the manuscript.

## FUNDING INFORMATION

This work was supported by a grant from the National Research Foundation of Korea (NRF‐2019R1A2C1009006 to Minhan Ka) and projects from the Korea Institute of Toxicology (1711195885 to Minhan Ka and 1711159829 to Sung‐Ae Hyun).

## CONFLICT OF INTEREST STATEMENT

The authors declare no conflicts of interest.

## ETHICS STATEMENT

All experimental procedures were approved by the Institutional Animal Care and Use Committee at the Korea Institute of Toxicology and met National Institutes of Health guidelines for the care and use of laboratory animals.

## Data Availability

The data that support the findings of this study are available from the corresponding author upon reasonable request.
